# Single-atom catalysts for high-energy rechargeable batteries

**DOI:** 10.1039/d1sc00716e

**Published:** 2021-05-17

**Authors:** Hao Tian, Ailing Song, Huajun Tian, Jian Liu, Guangjie Shao, Hao Liu, Guoxiu Wang

**Affiliations:** Centre for Clean Energy Technology, School of Mathematical and Physical Sciences, Faculty of Science, University of Technology Sydney Broadway Sydney NSW 2007 Australia hao.liu@uts.edu.au guoxiu.wang@uts.edu.au; State Key Laboratory of Metastable Materials Science and Technology, College of Environmental and Chemical Engineering, Yanshan University Qinhuangdao 066004 China shaoguangjie@ysu.edu.cn; Key Laboratory of Power Station Energy Transfer Conversion and System of MOE, School of Energy Power and Mechanical Engineering, North China Electric Power University Beijing 102206 China; State Key Laboratory of Catalysis, iChEM, Dalian Institute of Chemical Physics, Chinese Academy of Sciences 457 Zhongshan Road Dalian 116023 China; DICP-Surrey Joint Centre for Future Materials, Advanced Technology Institute, Department of Chemical and Process Engineering, University of Surrey Guildford Surrey GU2 7XH UK

## Abstract

Clean and sustainable electrochemical energy storage has attracted extensive attention. It remains a great challenge to achieve next-generation rechargeable battery systems with high energy density, good rate capability, excellent cycling stability, efficient active material utilization, and high coulombic efficiency. Many catalysts have been explored to promote electrochemical reactions during the charge and discharge process. Among reported catalysts, single-atom catalysts (SACs) have attracted extensive attention due to their maximum atom utilization efficiency, homogenous active centres, and unique reaction mechanisms. In this perspective, we summarize the recent advances of the synthesis methods for SACs and highlight the recent progress of SACs for a new generation of rechargeable batteries, including lithium/sodium metal batteries, lithium/sodium–sulfur batteries, lithium–oxygen batteries, and zinc–air batteries. The challenges and perspectives for the future development of SACs are discussed to shed light on the future research of SACs for boosting the performances of rechargeable batteries.

## Abstract 2

Single-atom catalysts are reviewed, aiming to achieve optimized properties to boost electrochemical performances of high-energy batteries.

## Graphical abstract

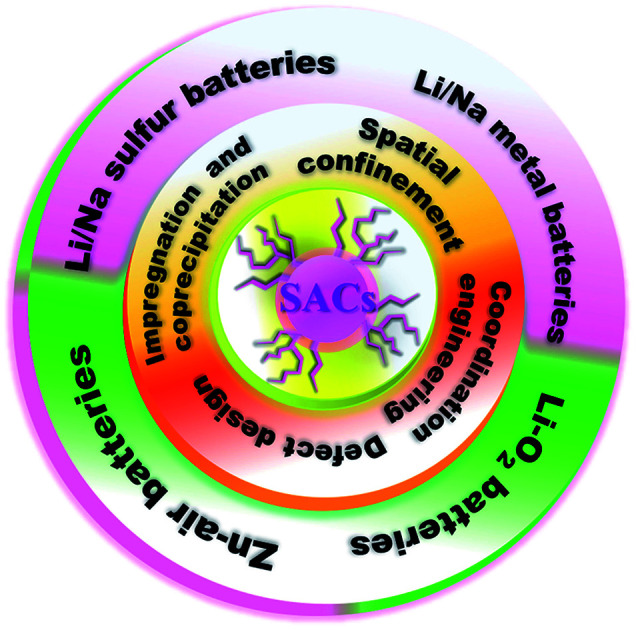

## 1. Introduction

Fossil fuels (natural gas, oil, and coal) play a crucial role in advancing the world's economy. The reliance on fossil fuels has invoked impending crises of future global energy needs and environmental damage, such as exhaustible natural resources, global warming, and pollution. It has become more urgent to develop renewable and environment-friendly electrochemical energy storage and conversion devices, such as lithium-ion batteries (LIBs), lithium–sulfur (Li–S) batteries, and metal–air batteries, in order to relieve our dependence on fossil fuels.^1^ However, low energy density, poor cycling stability, and high cost restrict the development of energy storage devices. Therefore, it is desired to design novel catalytic electrode materials to promote electrochemical reactions during the charge and discharge process, achieving high energy density and fast rate performances.

The design and synthesis of high-performance electrocatalysts are of great significance to achieve fast electrochemical reactions through reducing the energy barriers and accelerating the kinetics of the electrochemical process. Until now, substantial efforts have been devoted to achieving novel electrocatalysts with excellent performance through downsizing the electrocatalyst particles. The superior performances are generally attributed to the fact that the number of catalytically active sites can be increased, and the surface configuration and electronic structure can also be optimized.^2^ For example, metal-based nanoparticles and subnanoclusters have been successfully synthesized on matrix materials with large surface areas, such as carbon, g-C_3_N_4_, metal oxides, and metal sulfides, to obtain improved catalytic reactivity for various electrocatalytic applications.^3^ However, due to the high surface energy, the small and fine metal particles can be easily aggregated into clusters, leading to low catalytic activities.^4^ In addition, the intermediate conversion in the electrocatalytic process can be affected by the uneven size distribution of the catalysts, resulting in poor electrochemical performances. Therefore, it is essential to develop novel metal electrocatalysts with uniform size distribution and regular morphologies.

Single-atom catalysts (SACs), consisting of isolated metal atoms dispersed or anchored on matrix materials, have received tremendous attention in the field of catalysis and electrochemical systems.^4–8^ High activity and conversion efficiency for chemical reactions can be achieved using SACs with uniform and well-defined atomic dispersion. Compared with traditional catalysts, SACs display several merits in electrochemical energy storage and conversion, leading to excellent electrochemical performances with high energy density and high-rate performances for battery systems. First, energy barriers between chemical intermittents can be reduced due to the low-coordination and unsaturated active sites with high surface energy.^9^ Second, the charge transfer can be promoted through the strong bonding between matrices and single atoms.^10^ Lastly, soluble reaction intermittent can be immobilized by the strong polarity of SACs and the kinetic process in the electrochemical reactions can be accelerated.^11^ Therefore, the incorporation of SACs into electrode materials is a promising strategy to promote sluggish electrochemical reactions and increase the practical energy densities and rate capabilities.

SACs have been used to achieve high rate and long cycling performances for advanced battery systems, including lithium/sodium metal batteries, lithium/sodium–sulfur batteries, lithium–oxygen (Li–O_2_) batteries, and zinc–air (Zn–air) batteries. With the development of synthetic strategies, various SACs have been designed and synthesized to exploit the catalytic effects for enhancing the conversion kinetics, solving the challenges for the next-generation batteries. For example, the formation of lithium/sodium dendrite can be suppressed and the affinity between lithium/sodium metal and the support matrix can be improved by using SACs, leading to high coulombic efficiency and long cycling life.^12^ In addition, polysulfide migration can be restricted and redox reaction efficiencies can be enhanced for lithium/sodium–sulfur batteries. This is because SACs can catalytically improve polysulfide conversion and provide strong adsorption for soluble polysulfides.^11^ During the charge and discharge of Li–O_2_ and Zn–air batteries, the reaction kinetics of the oxygen reduction reaction (ORR) and oxygen evolution reaction (OER) can be accelerated.^13^

**Fig. 1:**
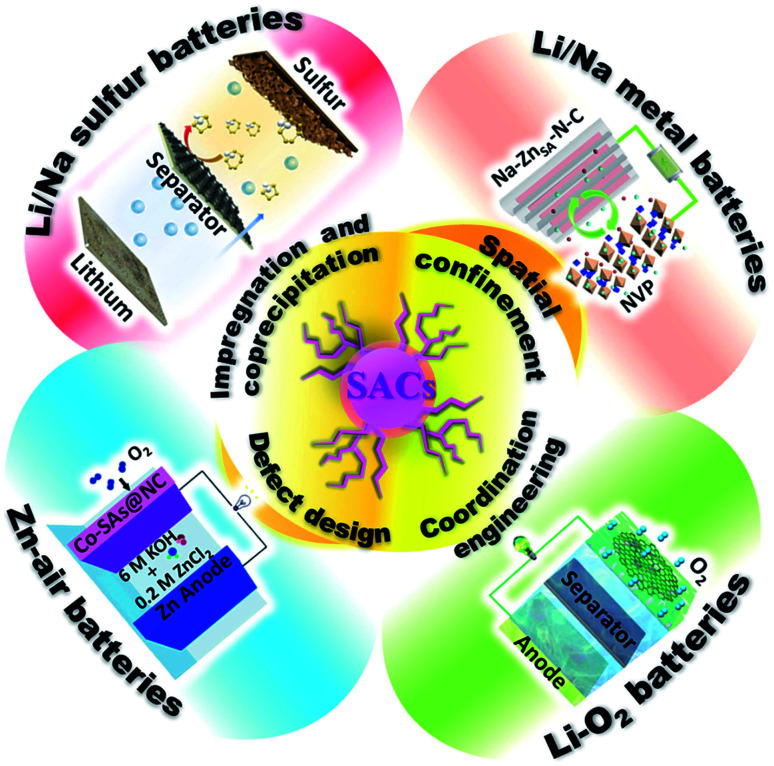
Schematic illustration of Single Atom Catalysts (SACs) synthesis strategies and their battery applications. Image for Li/Na metal batteries: reproduced by permission.^63^ Copyright 2019, American Chemical Society. Image for Li/Na sulfur batteries: reproduced by permission.^71^ Copyright 2019, American Chemical Society. Image for Li–O_2_ batteries: reproduced by permission.^84^ Copyright 2020, Springer Nature. Image for Zn–air batteries: reproduced by permission.^113^ Copyright 2019, Wiley-VCH.

Several reviews on SACs have been published in recent years, especially in the area of catalysis for conversion reactions.^14–17^ In this perspective, we systematically summarize the recent progress on SACs, mainly focusing on synthetic approaches to optimized properties of SACs for new generation high-energy rechargeable batteries (Fig. 1). The synthetic strategies for the preparation of SACs are summarized, and their applications in various electrochemical energy storage devices are presented. Then, we discuss the challenges and opportunities for the development of SACs for electrochemical energy storage.

## 2. Synthesis of isolated single-atom catalysts (SACs)

SACs with high catalytic activity, stability, and selectivity have become a new frontier in the field of catalysts.^2^ However, due to the high surface energy, single atoms within (or on the surface of) catalysts can easily aggregate into large nanoparticles, leading to inferior catalytic activity.^4^ To achieve highly active and stable SACs, the following requirements should be met: (i) homogeneous distribution of single metal atoms on the substrates; (ii) strong binding between the single atom metals and the support matrix; (iii) efficient mass transfer and electron transport; (iv) easily-accessible active sites.^18^ Therefore, the synthesis methods for SACs play an important role in achieving the above key criteria. In this section, we highlight various synthesis methods, including the creation of spatial confinement, generation defects on the matrix, impregnation or precipitation, and the construction of suitable coordination environments.

### 2.1. The spatial confinement method

To avoid aggregation into large nanoparticles, single atoms can be well-confined in the pore structures in or on the matrix. This spatial confinement synthesis method involves several steps: (i) the preparation of porous materials as matrices, including zeolites, metal–organic-frameworks (MOFs), and covalent-organic-frameworks (COFs); (ii) the incorporation of the metal precursors and the matrix; (iii) the carbonization of the formed materials in an inert atmosphere.

**Fig. 2:**
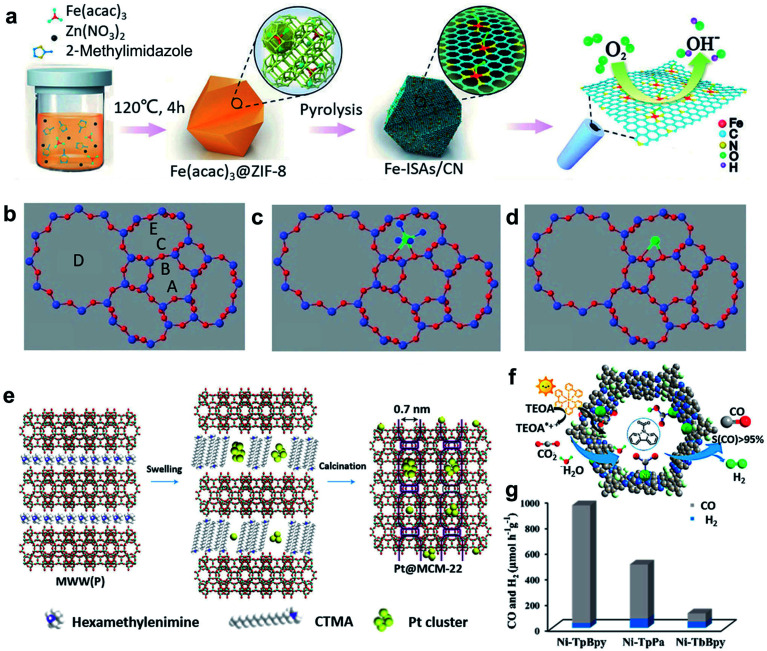
(a) Schematic illustration of the formation of Fe–ISAs/CN. Reproduced by permission.^22^ Copyright 2017, Wiley-VCH. (b–d) Models of zeolite LTL with pore nomenclature and [Pt(NH_3_)_4_]^2+^ and PtO_*x*_ located in the 8-membered ring at the C site. Reproduced by permission.^25^ Copyright 2014, Wiley-VCH. (e) Illustration of the preparation of Pt@MCM-22. Reproduced by permission.^26^ Copyright 2017, Springer Nature. (f) Schematic diagram of the photocatalytic selective reduction of CO_2_ over Ni-TpBpy. (g) Ni-TpBpy, Ni-TpPa, and Ni-TbBpy for the photocatalytic reduction of CO_2_ in a 2 h reaction. Reproduced by permission.^28^ Copyright 2019, American Chemical Society.

MOFs have been widely used as heterogeneous catalysts because of their large surface area, uniform pore size, and good chemical stability.^19^ Suitable mononuclear metal precursors can be encapsulated within the porous architecture through the pore confinement effect. Furthermore, abundant anchoring sites can be created *via* the modification of MOFs, ensuring the high dispersion and stability of single atoms.^20^ Zeolite imidazolate frameworks (ZIFs), one of the representative classes of MOFs, have attracted extensive attention, owing to their well-controlled morphology and porosity, large surface area, textural tunability, high thermal and chemical stability.^21^ For example, Li *et al.* reported the preparation of an isolated single-atom Fe/N-doped porous carbon (ISA Fe/CN) catalyst through the encapsulation of ZIF-8 and metal precursor Fe(acac)_3_, and the following calcination process (Fig. 2a).^22^ Because of the superior spatial confinement effect of the metal precursors from ZIF-8 and the abundant nitrogen species to stabilize the single metal atoms, the obtained ISA Fe/CN catalysts can achieve a high loading of Fe (about 2.2 wt%), resulting in excellent electrochemical performances. Apart from such pore-confinement calcination strategy, a solvent-assisted surface-integration strategy to obtain SACs was also introduced. Based on this strategy, Xing and colleagues successfully synthesized Cr/N/C SACs with highly dispersed Cr atoms and Cr–N_4_ coordination structure.^23^ When the catalysts were used for ORR, a low half-wave potential (0.773 V) and excellent durability were achieved.

Compared with MOFs, ZIFs provide a microporous aluminosilicate structure with abundant hydroxyl groups on the surface, which can serve as anchoring sites for single active sites.^24^ At the same time, the location of cationic sites in the zeolite framework can be substituted by ion exchange. After heat treatment, SACs with single atoms stabilized by adjacent oxygen centres can be obtained. For example, the pores of a Linde type “L” (“LTL”) zeolite were ion-exchanged to place K^+^ ions within the zeolite frameworks (producing a K-LTL zeolite), and then Pt metal precursors were incorporated to contribute Pt^2+^ ions (Fig. 2b–d). After oxidation at 633 K, Pt SACs with a well-defined structure and high dispersion of Pt single atoms can be acquired.^25^ On the other hand, another approach to achieve SACs based on zeolites is the *in situ* encapsulation of a metal complex and zeolite precursor with the following activation treatment.^26,27^ Because of the anionic nature of the aluminosilicate precursor, it is of great importance to foster a favourable interaction between the metal complex and aluminosilicate precursor when using this method. Corma *et al.* reported the generation of siliceous MCM-22 supported single Pt atoms and Pt clusters, starting from a siliceous layered precursor of MCM-22 with the incorporation with Pt metal complexes (Fig. 2e).^26^ It was found that the “cups” and “cages” of the MCM-22 zeolite could entrap single Pt atoms and small Pt clusters, which were stable within the MCM-22 framework even during high-temperature treatments.

Covalent organic frameworks (COFs), which are similar to MOFs, can also be used as support materials for the spatial confinement of single atoms.^28–30^ Owing to the abundant existence of B, N, and C elements, these heteroatoms can provide coordination sites for single metal atoms. For instance, single Ni sites can be covalently bonded within bipyridine ligands within the 2,2′-bipyridine-based COF (Fig. 2f).^28^ The obtained Ni-TpBpy SACs with ultrasmall size (0.17 nm) and high dispersion of Ni atoms proved to be an efficient photocatalyst for the selective reduction of CO_2_ to CO (Fig. 2g).

The spatial confinement method for the preparation of SACs is usually easy to achieve, and the obtained SACs have a homogeneous distribution of single atoms and well-defined structure. Nevertheless, this method requires further improvement. These include: (1) it usually involves high-temperature pyrolysis and rigorous reaction conditions to generate the micro-cages for trapping single atoms and to remove the ligands of mononuclear metal precursors, restricting the large-scale production; and (2) the spontaneous formation of micropores with small cavity dimensions can limit the mass transportation of the reactants.

### 2.2. Defect design method

Defect creation is another effective method to construct SACs capable of limiting the migration of single atoms on the matrix. The coordination of single metal atoms with the surrounding structure can be adjusted by defect generation, resulting in the formation of unsaturated coordination environments and vacancies. Thus, these defects can be treated as anchoring sites for the trapping and stabilization of single atoms. Many kinds of defects, such as anion vacancies, cation vacancies, and step edges, have been designed to prepare SACs.

**Fig. 3:**
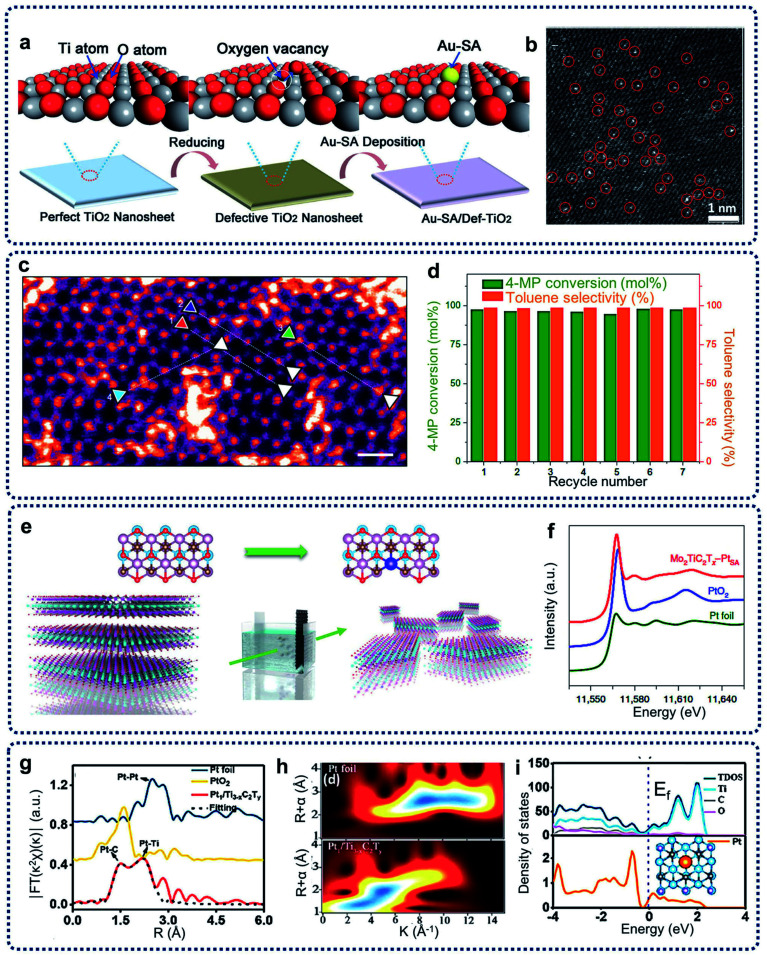
(a) Schematic illustration of the synthesis procedure and the results of Au-SA/Def-TiO_2_. (b) HAADF-STEM image, single atomic Au sites highlighted by red circles. Reproduced by permission.^32^ Copyright 2018, Wiley-VCH. (c) HAADF image of the basal plane of Co–SMoS_2_ (post-catalysis). Scale bar, 0.5 nm. (d) Stability test of Co–SMoS_2_ for HDO of 4-methylphenol. Reaction conditions: 3 MPa at 180 °C. Reproduced by permission.^33^ Copyright 2017, Springer Nature. (e) Schematic of the electrochemical exfoliation process of MXene with immobilized single Pt atoms. (f) Normalized XANES spectra at the Pt L_3_-edge of the Pt foil, PtO_2_ and Mo_2_TiC_2_T_*x*_–PtSA. Reproduced by permission.^34^ Copyright 2018, Springer Nature. (g) EXAFS FT *k*^2^-weighted *χ*(*k*) function spectra of Pt_1_/Ti_3−*x*_C_2_T_*y*_ and a reference. (h) WT for the *k*^2^-weighted Pt L_3_-edge EXAFS signals in Pt_1_/Ti_3−x_C_2_T_*y*_ and Pt foil. (i) TDOS and PDOS of Pt_1_/Ti_3−*x*_C_2_T_*y*_. Inset: schematic model. Reproduced by permission.^35^ Copyright 2019, American Chemical Society.

Inducing anion vacancies, including oxygen, sulfur, and nitrogen vacancies, have been commonly performed to create trapping sites to immobilize single atoms.^31^ Oxygen vacancies usually exist in oxides and hydroxides, such as CeO_2_, ZrO_2_, TiO_2_, Ca_10_(PO_4_)_6_(OH)_2_, Ni(OH)_2_ and Co(OH)_2_.^15^ For example, Wang *et al.* reported on oxygen-defective TiO_2_ nanosheet-supported single Au atom catalysts (Au-SA/Def-TiO_2_) with a tri-centered Ti–Au–Ti structure (Fig. 3a).^32^ As shown from the aberration-corrected high-angle-annular-dark-field scanning transmission electron microscopy (HAADF-STEM) image of Au-SA/Def-TiO_2_ in Fig. 3b, it was observed that large amounts of bright diffraction maxima were well dispersed across the nanosheet surface, revealing that the single Au sites were uniformly dispersed on the oxygen-defective TiO_2_ nanosheets. The Au single sites were effectively stabilized by the oxygen vacancies. The energy barrier of CO oxidation can be reduced, and the CO adsorption on the isolated Au sites can be optimized by the construction of such Ti–Au–Ti structures within the obtained catalysts. For the creation of sulfur and nitrogen vacancies, layered transition metal dichalcogenides, such as MoS_2_ and WS_2_, show abundant sulfur vacancies. In addition, TiN can provide enough nitrogen vacancies as anchoring sites for the single atoms. For example, through the engineering of sulfur vacancy distributions, single cobalt atoms can be successfully attached to monolayer MoS_2_, leading to the generation of Co–S–Mo active sites for the catalytic hydrodeoxygenation reaction at a low operating temperature (180 °C) (Fig. 3c and d).^33^ In addition, nitrogen vacancies can be induced to stabilize single Pt atomic sites on the TiN support surfaces, with the achieved catalysts showing excellent electrochemical performances.

Cation vacancies can also be created to restrict the migration of single atoms to prepare SACs, leading to the modification of electronic and coordination structures. Wang *et al.* also achieved the creation of Mo vacancies in transition metal MXene nanosheets with abundant exposed basal planes by electrochemical exfoliation method.^34^ As shown in Fig. 3e, single Pt atoms can be immobilized by Mo vacancies formed on the MXene nanosheets. Extended X-ray absorption fine structure spectroscopy (EXAFS) revealed that three carbon atoms coordinate with single-atomic-site Pt on the MXene nanosheets. X-ray absorption near-edge structure (XANES) in Fig. 3f indicated that the single Pt atoms in nanosheets are positively charged, confirming that the single-atoms of Pt were anchored in the Mo vacancy. Followed this work, single Pt atoms on two-dimensional Ti_3−*x*_C_2_T_*y*_ MXene nanosheets (Pt_1_/Ti_3−*x*_C_2_T_*y*_) were successfully achieved through a simultaneous self-reduction of the [PtCl_6_]^2−^ metal complexes at room temperature by abundant Ti-deficit vacancies.^35^ The Pt EXAFS Fourier transform curves of Pt_1_/Ti_3−*x*_C_2_T_*y*_ and references in Fig. 3g and a wavelet transform (WT) analysis of the Pt L_3_-edge EXAFS oscillations in Fig. 3h show the formation of the Pt–C and Pt–Ti bonds, and the existence of atomically dispersed Pt atoms in the prepared catalysts. The theoretical calculation in Fig. 3i reveals the strong electronic coupling of the Pt and C/Ti atoms, resulted in a downshifting of the d-band center compared to that of Pt NPs and optimal activation energy of the target reactants.

The preparation process in the defect design method usually involves the capture of a metal complex by the defects on the supports (such as metal oxide and carbon-based materials) and the post-treatment of the as-formed precursors through sintering. Metal oxide matrices have been proven to be effective catalyst supports for heterogeneous catalysis. The carbon-based SACs have shown superior electrochemical performances (such as in ORR) compared with state-of-art Pt/C catalysts. In addition, the metal loading of SACs can be tuned by changing the concentration of defects. Compared with metal oxide support materials, carbon-based support materials can achieve a higher metal content because of their large surface area. For example, Zhang *et al.* reported a catalyst comprising Ni single atoms supported on N-doped carbon nanotubes (Ni SAs/NCNTs). The specific surface area of the Ni SAs/NCNTs is 689 m^2^  g^−1^, and the loading of Ni is as high as 6.63 wt%.^36^ Compared with lower-dimensional materials, graphene has a larger theoretical specific surface area and is expected to obtain a higher single-atom loading. Jiang and co-workers achieved the synthesis of high Fe loading in iron single atoms on graphene (FeSA-G).^37^ The high loading of up to 7.7 wt% makes the FeSA-G have excellent ORR performance.

### 2.3. Impregnation and coprecipitation methods

**Fig. 4:**
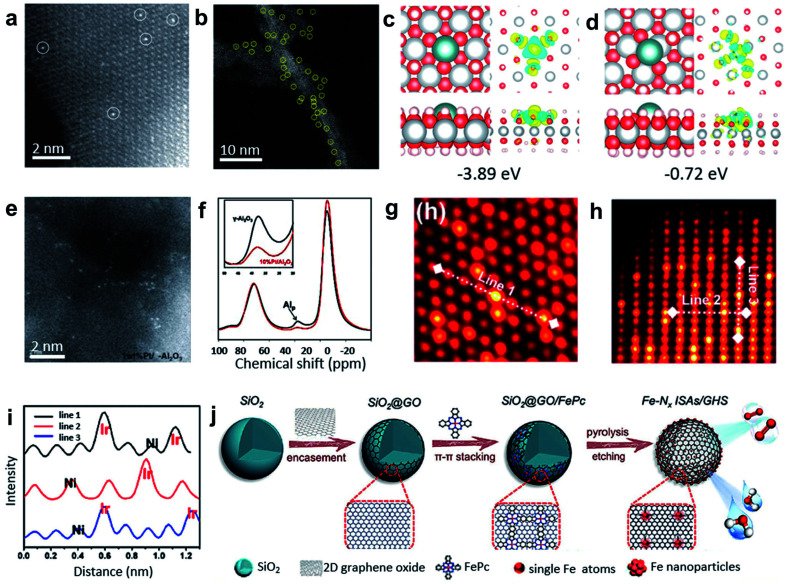
(a) HAADF-STEM images of Pt_1_/FeO_*x*_. Reproduced by permission.^38^ Copyright 2011, Springer Nature. (b) Representative AC HAADF-STEM image of the Pt_1_/Ni(OH)_*x*_ catalyst. The yellow circles were drawn around SAS Pt. (c and d) Studies of the interaction between the Ni^2+^ vacancies and isolated Pt atoms. Top and side views of the most stable structure and charge density difference for the Pt atom adsorbed on the Ni(OH)_2_ with Ni^2+^ vacancies (c) and without Ni^2+^ vacancies (d). The cyan, gray, red, and white balls refer to the Pt, Ni, O, and H atoms, respectively. For charge density difference, yellow (blue) corresponds to the charge accumulation (depletion) plotted with an isovalue of ±0.01 e Å^−3^. Reproduced by permission.^40^ Copyright 2018, Springer Nature. (e) High-resolution STEM images of 1 wt% Pt/γ-Al_2_O_3_. (f) The ^27^Al MAS-NMR spectra of γ-Al_2_O_3_ (black) and 10 wt% Pt/γ-Al_2_O_3_ (red) (both samples were calcined at 573 K before NMR measurements). Reproduced by permission.^41^ Copyright 2009, the American Association for the Advancement of Science. (g and h) Atomic STEM images along (g) the [111] zone axis and (h) [211] zone axis. (i) The lines represent the line profiles for the HAADF intensity analysis labeled in (g) and (h). Reproduced by permission.^42^ Copyright 2020, American Chemistry Society. (j) FePc molecules supported on 3D graphene hollow nanospheres facilitating the formation of the Fe single atom arrays. Reproduced by permission.^43^ Copyright 2019, Wiley-VCH.

Impregnation and coprecipitation methods have been used to create defects and unsaturated sites for synthesizing SACs. The differences in the electronic effects and bonding between the metal precursors and the supports lead to the dispersion of single metal atoms within the catalysts. Foremost, Zhang and co-workers used the coprecipitation method to prepare the Pt_1_/FeO_*x*_ SACs with only 0.17 wt% Pt loading (see Fig. 4a).^38^ Theoretical calculations indicated that the Pt single atoms were anchored at three-fold hollow sites on the O_3_-terminated surfaces of the FeO_*x*_ supports. This method was successfully extended further to prepare Ir_1_/FeO_*x*_ SACs with a mere 0.01 wt% Ir loading.^39^ Additionally, impregnation and coprecipitation approaches have been further applied to prepare SACs with higher loadings. For example, Wang and co-workers reported on nickel hydroxide (Ni(OH)_*x*_) nanoboards with abundant Ni^2+^ vacancy defects as supports to achieve Pt_1_/Ni(OH)_*x*_ SACs with Pt loading up to 2.3 wt% by an impregnation method (Fig. 4d).^40^ As shown in Fig. 4b and c, density functional theory (DFT) calculations verified the lower formation energies of the isolated Pt atoms loaded on the defective Ni(OH)_2_ with Ni^2+^ vacancies compared with the common perfect Ni(OH)_2_. In the synthesis of various alkynes and alkenes, the Pt_1_/Ni(OH)_*x*_ catalyst displayed excellent activity and selectivity.

Nonreducible oxides, such as Al_2_O_3_, can also anchor single-atom metal species at unsaturated centres. Kwak and co-workers impregnated γ-Al_2_O_3_ with aqueous Pt(NH_3_)_4_(NO_3_)_2_ solutions, and then calcined at 573 K to achieve Pt/γ-Al_2_O_3_ SACs with a Pt loading of 1.0 wt% (see Fig. 4e).^41^ The results of ultrahigh magnetic field Al magic-angle spinning (MAS) nuclear magnetic resonance (NMR) spectroscopy (Fig. 4f) and HAADF-STEM characterization indicated that pentacoordinated Al^3+^ centres on the γ-Al_2_O_3_ surface were the anchoring sites for the precursor of metallic Pt and PtO. The existence of unsaturated centres of γ-Al_2_O_3_ was the key to effectively anchoring the Pt single atoms. It was also demonstrated that particles could migrate on the surface of the α-Al_2_O_3_ matrix without the pentacoordinated Al^3+^ sites. NiO can also be used as a support to prepare SACs. NiO-grown carbon cloth (CC) was first impregnated with chloroiridic acid ethanol solution, and then calcined at 350 °C for 20 min to obtain the Ir–NiO/CC catalyst with Ir loading up to ∼18 wt%.^42^ STEM and EXAFS results demonstrated the formation of the six-coordinated Ir–O between the isolated Ir atoms on the NiO substrates (Fig. 4g–i), which resulted in a high loading of Ir single atoms in the catalysts. Moreover, this method was extended to prepare single Mn, Fe, Co, Ru, and Pt atoms supported on NiO. In addition, the π–π conjugation effect can be used to anchor single atoms through both impregnation and coprecipitation methods.^44,45^ Xu and co-workers fabricated Fe–N_*x*_ ISAs/GHSs catalysts by coating GO nanosheets on SiO_2_ nanospheres (SiO_2_@GO) with subsequent annealing of the mixture of SiO_2_@GO and FePc in an N_2_ atmosphere for 3 h and an etching process (Fig. 4j).^43^ The as-prepared SACs exhibited an outstanding *E*_1/2_ of 0.87 V (*vs.* RHE) in ORR.

Enormous efforts have been devoted to preparing SACs *via* defect engineering through impregnation and coprecipitation methods to achieve unsaturated site controlling and π–π conjugation anchoring. The strong interactions between the single atoms and their support structures can effectively facilitate the successful synthesis of SACs. The impregnation and coprecipitation methods are based on a simple wet chemical strategy, and the metal loading can be controlled by the amounts of defects, unsaturated sites, and overall surface areas. Therefore, to achieve SACs with high loading, a combination of effects is required for the generation of abundant defects, unsaturated sites, and a large surface area.

### 2.4. Coordination engineering method

The coordination engineering method to achieve SACs takes advantage of the bond formation between single metal atoms and the coordination atoms to immobilize and stabilize single atoms. Until now, precursors such as MOFs, graphdiyne, and C_3_N_4_ have been utilized to prepare SACs. Heteroatoms with lone pair electrons such as N, P, and S in the precursors can provide coordination sites to adsorb and anchor single metal atoms.

**Fig. 5:**
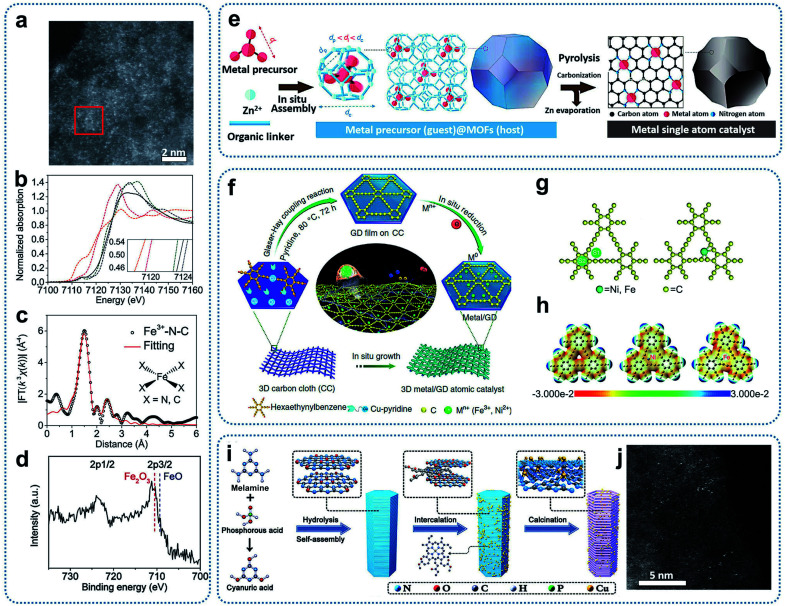
(a) Aberration-corrected HAADF-STEM image of Fe^3+^–N–C. (b) Fe K-edge XANES spectra of Fe^3+^–N–C (black), Fe_2_O_3_ (blue dashed), Fe^3+^ TPPCl (green dashed), FeO (pink dashed), and Fe foil (orange dashed). (Inset) Enlargement of the main edges. (c) *R*-space Fe K-edge EXAFS spectra. Shown are data (black) and fitting curves (red). (d) Fe 2p XPS spectrum. Reproduced by permission.^46^ Copyright 2019, the American Association for the Advancement of Science. (e) A host–guest strategy for the fabrication of metal single-atom catalysts. Reproduced by permission.^47^ Copyright 2020, Springer Nature. (f) Illustration of the synthesis of Ni/GD and Fe/GD. (g) Adsorption of single metal atoms on GD (left: possible adsorption sites; right: optimized configuration). (h) Electrostatic potential maps of pristine GD, Ni/GD, and Fe/GD, respectively. Reproduced by permission.^49^ Copyright 2018, Springer Nature. (i) Schematic illustration of the preparation of SA–Cu–TCN. (j) Representative HAADF-STEM image of SA–Cu–TCN. Reproduced by permission.^50^ Copyright 2020, Wiley-VCH.

Fe–N–C catalysts with atomically dispersed Fe sites and an overall Fe loading of about 2.8 wt% were synthesized through the thermalization of MOF precursors (Fe-doped ZIF-8).^46^ Zinc ions in the ZIF-8 precursor could be reduced and then evaporated during pyrolysis. Aberration-corrected HAADF-STEM images of the resultant material presented bright spots, corresponding to the atomically dispersed Fe and Zn sites (Fig. 5a). Fe K-edge XANES, EXAFS, and XPS spectra, as shown in Fig. 5b–d, indicate a planar Fe–X_4_ (X = N or C) structure with a +3 oxidation state of Fe ions in the Fe–N–C catalysts. In addition, Li and co-workers developed a general host–guest synthesis strategy through coordination engineering to prepare high loading SACs supported on nitrogen-doped carbon (M_1_/CN, M = Pt, Ir, Pd, Ru, Mo, Ga, Cu, Ni, Mn).^47^ Under the condition of *in situ* crystal growth, the metal precursors were encapsulated in ZIFs cages to form a uniformly dispersed host–guest structure. In the subsequent high-temperature calcinations, the metal precursors were decomposed and immobilized on the nitrogen-doped carbon substrates (Fig. 5e). The method has considerable universality and could achieve the synthesis of various SACs, covering 3d, 4p, 4d and 5d catalytically active elements from the periodic table. For Ir_1_/CN catalysts with an Ir loading of 1.2 wt%, XANES results indicated the presence of stable forms of Ir–N_4_.

Graphdiyne is another kind of precursor that can tightly immobilize single metal atoms through Metal–C bonds. For example, graphdiyne was employed to adsorb Fe^3+^ ions, followed by reduction with NaBH_4_ to prepare single-atom Fe–graphdiyne catalysts.^48^ Li and co-workers also prepared graphdiyne grown on the surface of three-dimensional carbon cloth, which can be used as supports to achieve SACs with Fe or Ni single atoms stabilized on a graphdiyne surface (Ni/GD, Fe/GD) through the electrochemical reduction method (Fig. 5f).^49^ From the theoretical calculations, both of the optimization results of the calculation models showed that the single metal atoms were bonded to the carbon atoms of the initial butadiyne (Fig. 5g and h). This bonding was different from that observed in SACs with metal–N coordination, which were obtained from MOF precursors.

Graphitic carbon nitride (g-C_3_N_4_) with six-fold cavities connected by tertiary amines possesses plentiful nitrogen coordinators, which can be utilized to capture single metal atoms. For example, chlorophyll sodium copper was first incorporated into melamine-based supramolecular precursor layers. After thermal polymerization, Cu-based SACs were achieved (Fig. 5i and j).^50^ XANES spectra and theoretical calculations indicated that each Cu atom could coordinate with three in-plane N atoms or four N atoms of the two neighbouring C_3_N_4_ layers, forming two different types of Cu–N_*x*_ as effective charge transport channels. This work demonstrated that the dense six-fold cavities based on g-C_3_N_4_ are effective to anchor single metal atoms to obtain SACs.

Compared to the previously discussed methods (spatial confinement, defect design, and impregnation & coprecipitation), the coordination engineering method can control the coordination structure, the number of anchored metal atoms, and the interaction between metal atoms and the ligands by changing the synthesis parameters. Moreover, the versatility in selecting the adjustable construction units of the precursor materials facilitates the synthesis of various SACs with well-controlled structures and functionalities.

### 2.5. Other specialized methods (atomic layer deposition method, photochemical method, freezing-assisted method, microwave-assisted method, and ball-milling method)

In addition to the abovementioned methods, other methods used to prepare SACs have been implemented. These include atomic layer deposition (ALD), photochemical, freezing-assisted, microwave-assisted, and a ball-milling method. ALD is a powerful technique for precisely achieving targeted SACs, in which the matrix materials may be alternately exposed to pulsed vapor clouds or jets of different metal precursors, and single metal atoms are deposited on the surface of the support in a self-limiting manner. To obtain SACs with uniform geometrical structures, Sun and co-workers first synthesized atomic-layer deposited Pt single atoms on graphene nanosheets (Pt/graphene) by using (methylcyclopentadienyl)-trimethylplatinum (MeCpPtMe_3_, purity 98%) as the deposition source, and moderated the deposition density of Pt by controlling the ALD cycles.^51^ After the ALD process for 50 cycles, single atomic dispersion was achieved on the surface of graphene. However, this technique is often constrained by expensive experimental equipment and low yield.

The photochemical method is a mild and simple preparation method, and was first utilized for the preparation of SACs in 2015 by Zheng and co-workers.^52^ In this synthesis route, the key to the formation of single atoms was the adsorption of H_2_PdCl_4_ on TiO_2_ with subsequent exposure of the solution to a UV lamp. The loading of Pd in the prepared Pd_1_/TiO_2_ can be controlled to 1.5 wt%. To control the atomic diffusion of metal ions in the solution, near-freezing temperature control technology has been introduced to this photochemical method. For example, the ice solution containing H_2_PtCl_6_ was first irradiated with a UV lamp in a lyophilizer to obtain an ice solution with Pt single atoms, and subsequently impregnated with mesoporous carbon to obtain Pt_1_/MC.^53^ Furthermore, such a freezing-assisted strategy can be combined with wet chemical methods for ion-adsorption to synthesize atomic SACs, which can effectively restrict the random diffusion and aggregation of metal precursors. In a typical example, a solution of GO dispersion containing Ni^2+^ and Y^3+^ ions were first freeze-dried, and then calcined to obtain the Ni/Y_2_O_3_ nanosheets with single Ni atoms.^54^

The microwave-assisted method provides another strategy that can quickly initiate a chemical reaction to achieve single atom dispersion. Microwave-assisted methods have many advantages for the SACs synthesis, such as simple and fast reaction approach, reduced reaction times, environmentally friendliness, and efficient atom utilization. For example, microwave heating has been used to achieve high temperatures with the simultaneous reduction of graphene oxide, nitrogen doping, and embedding of metal atoms into a graphene lattice in a one-step process.^55^ An impressive aberration-corrected HAADF-STEM image of the prepared SACs “Co-NG-MW” shows a uniform dispersion of bright dots, which were attributed to the single atoms of cobalt.

The ball milling method based on demonstrated mechanochemical processes has inherent benefits, such as easy large-scale production and low solvent consumption. Kilogram-level SACs have been prepared *via* ball milling by Ma and co-workers.^56^ Palladium acetylacetonate and zinc acetylacetonate were first ball milled, followed by calcination in an air atmosphere at 400 °C for 2 hours to obtain Pd_1_/ZnO SACs at a fabrication scale of 1 kg batches of the final product. Across different batch preparation size-scales, the obtained catalysts had the same chemical structure and catalytic performance for the hydrogenation of phenylacetylene. This work indicates that the ball milling method has great potential for the mass production of SACs.

## 3. Applications of SACs in rechargeable batteries

SACs have significant advantages for various rechargeable battery systems because of their unique coordination characteristics and active sites. For lithium/sodium–metal batteries, SACs can potentially provide unique lithium/sodium nucleation sites, suppress dendrite formation, and enhance metal affinity, thereby achieving high coulombic efficiency and long cycling life. Moreover, SACs could suppress polysulfide migration and improve the redox reaction efficiencies in lithium/sodium–sulfur batteries due to their strong affinity for soluble polysulfides and the catalytical enhancement of polysulfide conversion to the final product. SACs can also promote electrocatalytic reactions, such as the oxygen evolution reaction and oxygen reduction reaction, improving the electrochemical performances and stability of lithium–oxygen and zinc–air batteries. In this section, we will summarize and discuss the rational design of SACs with optimal structure and chemical composition, and their applications in various types of advanced energy storage devices.

### 3.1. Lithium/sodium metal batteries

Lithium metal batteries have been extensively studied in the past decades because of their high theoretical specific capacity (3860 mA h g^−1^) and the lowest electrochemical potential (−3.04 V *vs.* standard hydrogen electrode) of lithium metal anodes.^57^ Unfortunately, the use of untreated Li–metal electrodes is prone to the growth of Li dendrites, large electrode volume change and production of an unstable interface during discharging–charging processes. These effects result because of the high activity of lithium metal and may lead to internal short circuits, low coulombic efficiencies, and shortened cycle ability.^58,59^ To solve these issues, remarkable efforts have been devoted to electrolyte additives, employing solid electrolyte interphase layers, and engineering electrodes for dendrite prevention and suppression.^60^

**Fig. 6:**
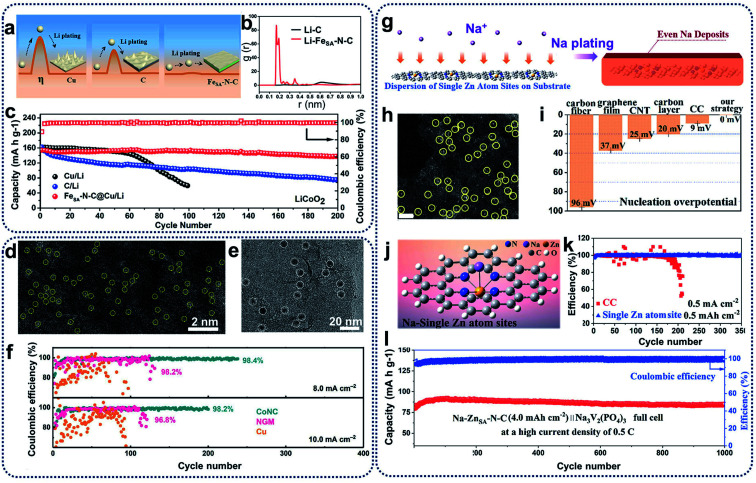
(a) Schematic representation of the Li plating process on the Cu, C@Cu, and Fe_SA_–N–C@Cu electrodes (*η* means the nucleus overpotential of the Li deposition). (b) The radial distribution function (RDFs) of Li–C and Li–Fe_SA_–N–C. (c) Cycling performances of full cells with LiCoO_2_ as the cathode and Fe_SA_–N–C/Li (C/Li, Cu/Li) as the anode at 1C (1C = 274 mA g^−1^). Reproduced by permission.^61^ Copyright 2019, American Chemical Society. (d) HAADF-STEM image of the CoNC materials. (e) TEM image of the Li nucleation sites on CoNC at 0.1 mA cm^−2^ for 5 min. (f) Long-term electrochemical cycling performance of CoNC, NGM, and Cu electrodes at a fixed capacity of 8.0 mA h cm^−2^ and 10.0 mA h cm^−2^, respectively. Reproduced by permission.^62^ Copyright 2019, Wiley-VCH. (g) Schematic illustration of the Na plating behavior on the Zn_SA_−N–C electrodes. (h) HAADF-STEM image of Zn_SA_−N–C. Single Zn atoms are highlighted in yellow circles. Scale bar, 2 nm. (i) The nucleation overpotential of different substrates. (j) DFT calculations on the affinity between carbon and a single Zn atom site to evaluate the strong interaction for Na ions. Na is expected to nucleate from single Zn atom sites. (k) Coulombic efficiency with an areal capacity of 0.5 mA h cm^−2^ at a current density of 0.5 mA cm^−2^. (l) Long-term cycling stability with high coulombic efficiency of almost 100% at 0.5C. Reproduced by permission.^63^ Copyright 2019, American Chemical Society.

Recent studies showed that the introduction of heterogeneous seeds within a carbon matrix to be used as a lithiated electrode can affect the distribution and growth of dendrites, and reduce the nucleation overpotential.^12^ One of the most effective methods is to fabricate SACs to suppress lithium dendrites and enhance the affinity between lithium and host materials. For example, single iron atoms in an N-doped carbon matrix (Fe_SA_–N–C) have been prepared as lithiophilic sites for the minimization of Li nucleation.^61^ Fe_SA_–N–C catalysts present a lower overpotential (0.8 mV) compared with pristine carbon matrix (18.6 mV) because single-atom Fe- and N-doping of a carbon matrix can lead to the uniform deposition of lithium on the electrode surface, and restrict the growth of lithium dendrites (Fig. 6a). Theoretical calculations also showed the excellent affinity between Li ions and Fe_SA_–N–C catalysts at the atomic level (Fig. 6b). As a result, while bare Cu foil and C@Cu electrodes present unstable Coulombic Efficiency (CE), the Fe_SA_–N–C@Cu electrode could achieve a CE of 98.8% for about 200 cycles (Fig. 6c), indicating that Fe_SA_–N–C catalysts can enhance the lithium utilization and suppress the growth of Li dendrites during electrochemical processes in lithium metal batteries. Apart from the single Fe atom for lithium–metal batteries, Zhang *et al.* reported the synthesis of atomically dispersed CoN_*x*_-doped graphene (CoNC) with 0.40 wt% Co to accommodate dendrite-free lithium deposition.^62^ In the HAADF-STEM image reproduced in Fig. 6d, uniformly distributed Co atoms (highlighted by yellow circles) can be observed. As shown in Fig. 6e (visible as dense black spots), uniform lithium nucleation sites were deposited on carbon supports. The existence of atomically dispersed Co atoms and N dopants within a graphene framework can tune the local electronic structure, and facilitate the adsorption of lithium ions and the subsequent nucleation process. In addition, large volume changes can be alleviated during reversible Li plating/stripping processes, resulting in stable SEI generation. Consequently, CoNC anodes retained a high and stable CE of 98.4% and 98.2% for over 200 cycles at 8.0 and 10.0 mA cm^−2^, respectively (Fig. 6f). CoNC catalysts exhibited superior electrochemical performances above nitrogen-doped graphene or Cu, further confirming that CoNC electrodes demonstrate strong lithiophilicity and high reversibility as Li metal anodes during repeated plating/stripping.

Compared with lithium metal batteries, sodium–metal batteries with high theoretical specific capacity (1166 mA h g^−1^) and low electrochemical potential (−2.714 V *vs.* standard hydrogen electrode) have received great attention due to the low price and wide availability of sodium sources.^64^ Like lithium anodes, sodium anodes also suffer from high reactivity of sodium metal, huge volume change effect, uneven sodium deposition, and dendrite growth during the plating and stripping processes.^65^ Moreover, because the radius of sodium ions is larger than that of lithium ions, the low utilization of sodium metal and the uncontrollability of sodium dendrites are more serious, leading to poor cycling ability and low reversibility.^66^ To address these problems, strategies such as constructing artificial SEI layers, designing electrolyte additives, and adding doping heteroatoms have been utilized, which are similar to the techniques used in lithium metal batteries. Recently, SACs have also been introduced for sodium dendrite suppression and sodium affinity enhancement for sodium metal batteries. The Yan group proposed a novel synthetic strategy to prepare N-anchored single Zn atoms on carbon supports (Zn_SA_–N–C) through direct calcination of Zn-containing ZIFs, maximizing sodium utilization and regulating sodium deposition (Fig. 6g).^63^ Highly isolated atomic Zn species (highlighted as yellow circles in Fig. 6h) have been homogeneously distributed on carbon supports. The nucleation overpotential of metallic Na deposition on Zn_SA_–N–C was much lower than that on carbon cloth (CC) and bare Cu foil because of the thermodynamic mismatch between the Zn_SA_–N–C host and sodium metal (Fig. 6i), indicating that the heterogeneous nucleation barriers can be effectively overcome through the use of Zn_SA_–N–C catalysts. DFT calculations further showed a large binding energy (0.34 eV) between the single Zn atoms and sodium ions, revealing that single zinc atoms could serve as nucleation centres for sodium ions to spatially control and suppress the growth of sodium dendrites (Fig. 6j). As shown in Fig. 6k, test cells with Zn_SA_–N–C anodes displayed a high CE of 99.8% over 350 cycles. Moreover, when assembled with the Na_3_V_2_(PO_4_)_3_ cathodes in full cells (Fig. 6l), single Zn atom-decorated anodes exhibited excellent long-term cycling performances for 1000 cycles with a high CE of 100% at the current density of 0.5C. Single Zn atoms in the Zn_SA_–N–C anodes can enhance the surface activity, reduce the reaction energy barrier, and accelerate the adsorption of sodium ions in the reversible sodium stripping/plating processes, leading to stable long-term cycling behaviours.

### 3.2. Lithium/sodium–sulfur batteries

Rechargeable lithium-ion batteries (LIBs) have become popular and pervasive as sustainable energy storage devices due to their long cycle life, high specific power, and energy density.^67^ However, current LIBs have an energy density of less than 200 W h kg^−1^ or 600 W h L^−1^, which cannot meet the ever-increasing demands from many emerging applications, such as electric vehicles.^68^ To achieve high energy density, alternative rechargeable batteries have been developed and widely investigated in recent years. Of these, lithium–sulfur (Li–S) and sodium–sulfur (Na–S) batteries have attracted growing attention due to several advantages, such as the natural abundance of sulfur, high specific energy density (2600 W h kg^−1^), and high theoretical capacity (1675 mA h g^−1^).^68^ The overall redox reactions involve the formation of soluble and insoluble polysulfide intermediates. The “shuttle effect” caused by the migration of electrolyte-soluble polysulfides to the anode is one of the most important issues that hinders the electrochemical performance of Li–S and Na–S batteries. In addition, the chemical reactions during discharging and charging consist of the sulfur molecule ring openings and closings, chain breaking and reforming of polysulfide intermediates, and the bonding and debonding of polysulfide intermediates, leading to complicated and sluggish reaction kinetics. Consequently, the practical applications of Li–S and Na–S batteries have been hindered by fundamental challenges, including the insulating nature of sulfur, the dissolution and migration of polysulfide intermediates, and the volume expansion of sulfur upon lithiation/sodiation, leading to performance deterioration with low sulfur utilization, low coulombic efficiency, and fast capacity decay.^69^ So far, various strategies have been proposed to improve the electrochemical performance of sulfur cathodes. One of the common methods is to incorporate sulfur particles within electronically conductive materials; for example, encapsulating sulfur particles within a porous carbon matrix to suppress the diffusion and impede the shuttle effect of polysulfide intermediates. Another effective strategy is to incorporate transition or noble metal particles, metal sulfides, and single atoms into the electrode materials to accelerate redox reactions and achieve higher sulfur utilization. Various SACs, such as carbon materials with Fe, Co, Zn, and Ni single atoms, have been prepared for cathodes and separators for Li–S and RT-Na–S batteries.^11,70–72^ By promoting polysulfide conversion, SACs can effectively decrease the polarization and suppress shuttle effects, leading to the enhancement of rate and cycling performance.

**Fig. 7:**
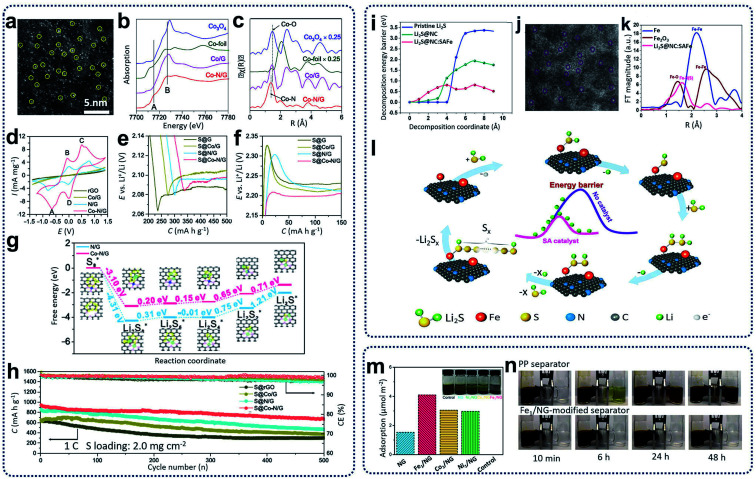
(a) HAADF-STEM images of Co–N/G. (b) XANES and (c) FT-EXAFS in *R* space for Co–N/G and reference samples including Co/G, Co-foil, and Co_3_O_4_. (d) CVs of symmetric cells with Co–N/G, N/G, Co/G, and rGO electrodes. (e) Discharge and (f) charge profiles of S@Co–N/G, S@N/G, S@Co/G, and S@rGO electrodes showing the overpotentials for conversion between soluble LiPSs and insoluble Li_2_S_2_/Li_2_S. (g) Energy profiles for the reduction of LiPSs on N/G and Co–N/G substrates. (Insets) The optimized adsorption conformations of the intermediate species on the N/G and Co–N/G substrate. (h) Cycling performance of S@Co–N/G, S@N/G, S@Co/G, and S@rGO electrodes. Reproduced by permission.^11^ Copyright 2019, American Chemical Society. (i) Comparison of the delithiation energy barriers of the pristine Li_2_S and the Li_2_S@NC with/without the SAFe catalyst. (j) HAADF-STEM images of single iron atoms in a Li_2_S@NC:SAFe nanocomposite. (k) Fourier transformed curves of the Fe K-edge EXAFS spectra of Li_2_S@NC:SAFe, Fe metal foil, and Fe_2_O_3_. (l) A proposed mechanism for the SAFe catalyzed Li_2_S delithiation reaction. Reproduced by permission.^70^ Copyright 2019, Elsevier. (m) Electrochemical titrations of Li_2_S_6_ adsorption on NG and M_1_/NG in 1,3-dioxolane (DOL)/1,2-dimethoxyethane (DME). Inset: digital photo of the Li_2_S_6_ solution after 12 h. (n) Polysulfide permeation tests for the Fe_1_/NG-modified separator. Reproduced by permission.^71^ Copyright 2019, American Chemical Society.

SACs carbon materials as sulfur hosts have been synthesized for the improvement of the electrochemical performance of Li–S batteries. For example, single Co atoms incorporated in N-doped graphene (Co–N/G) have been obtained through heat treatment of a mixture of graphene oxide and cobalt chloride under ammonia and argon gas at 750 °C. These have been used as cathode materials (S@Co–N/G) for Li–S batteries.^11^ As shown in the HAADF-STEM image in Fig. 7a, Co atoms were verified to be homogeneously dispersed on the graphene supports. X-ray absorption spectroscopy (XAS) spectra, as shown in Fig. 7b and c, reveal the formation of Co–N–C coordination moieties. To understand the catalytic effect of the Co–N–C centers incorporated in graphene supports, cyclic voltammetry profiles, as shown in Fig. 7d, indicate that S@Co–N/G materials show pronounced cathodic and anodic peaks. The discharge and charge profiles in Fig. 7e and f indicate that S@Co–N/G presented the lowest overpotentials, revealing the high catalytic effects of Co–N/G materials for the conversion of lithium polysulfides. Further theoretical calculations in Fig. 7g were used to investigate the improvement of the reaction kinetics during the discharging and charging processes, revealing that the reaction activation energies were greatly reduced, and the sulfur reductions were more thermodynamically favorable on Co–N/G supports. When used as cathode materials for Li–S batteries, the as-prepared cathodes containing 90 wt% sulfur loading retained a discharge capacity of 681 mA h g^−1^ with coulombic efficiency of ∼99.6% and an average capacity decay rate of 0.053% per cycle (Fig. 7h). In further work, Zhang *et al.* also designed and synthesized cathode materials containing L_2_S and single Fe atom catalysts supported on porous nitrogen-doped carbon materials (Li_2_S@NC:SAFe) through the calcination of the mixture, including polyaniline, lithium sulfides, and iron acetate precursors, at 700 °C under argon atmosphere.^70^ Theoretical calculations, as summarized in Fig. 7i, show that the energy barriers for the delithiation of Li_2_S can be decreased by using highly active single iron atom catalysts, facilitating Li ion transportation. The HAADF-STEM image in Fig. 7j clearly shows a homogenous distribution of single Fe atoms, which is also confirmed from the EXAFS results shown in Fig. 7k. Based on the experimental results and theoretical calculations, a mechanism was proposed for the utilization of single iron catalysts for Li–S batteries (Fig. 7l). First, Li–S bond lengths will be increased by coordination between SACs and Li_2_S. During the charging process, lithium ions could be easily decoupled from the intermediate [N–F⋯S–Li_2_], which could incorporate with neighboring Li_2_S molecules to generate a polysulfide through repeated delithiation processes. When tested as Li–S batteries, Zhang's as-prepared cathode materials exhibited high-rate performance (588 mA h g^−1^ at 12C) and excellent cycling capability with low capacity fading (0.06% per cycle for 1000 cycles at 5C). Moreover, SACs can also be used as separator layers for Li–S batteries. Xie and colleagues prepared separators for Li–S batteries through coating graphene foam impregnated with SACs on commercial polypropylenes.^71^ Synthesis of the catalyst (Fe_1_/NG) was achieved *via* heat treatment of GO foams containing the FeCl_3_ precursor at 750 °C under Ar/NH_3_ atmosphere. As shown in Fig. 7m, the Fe_1_/NG catalysts showed strong adsorption ability for polysulfides. After the Fe_1_/NG catalysts were coated on commercial separators, it was observed that no polysulfides diffused through the Fe_1_/NG-modified separators after 48 h (Fig. 7n) compared with significant leakage through the plain commercial separators. This indicates that the Fe_1_/NG catalysts can immobilize lithium polysulfides through strong electrostatic capability between metal and non-metal atoms, leading to the minimization of the shuttling effect and an overall improvement in the cell performance.

**Fig. 8:**
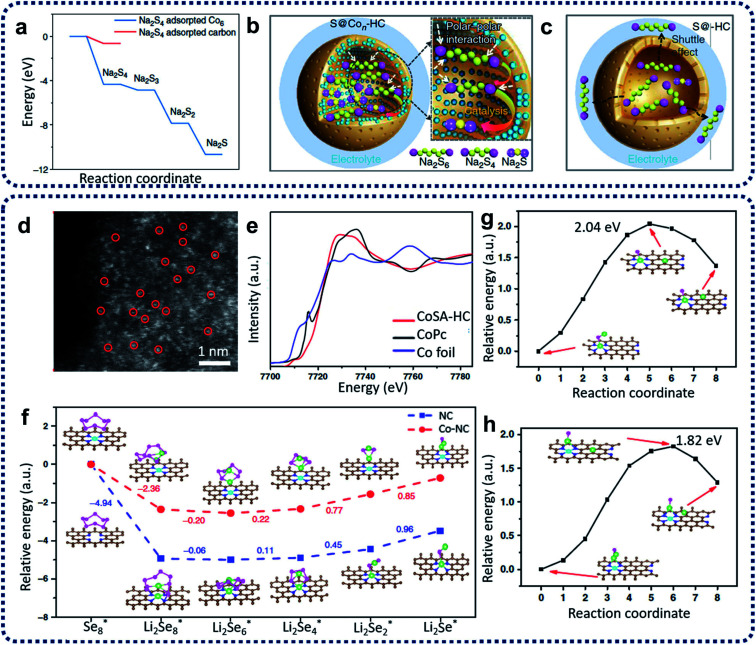
(a) Energy profiles of the Na_2_S_4_ adsorption on the carbon-supported Co_6_ cluster (in blue) and carbon support (in red). (b) Schematic illustrations of the electrode reaction mechanism of the atomic cobalt-decorated hollow carbon sulfur host (S@Co_*n*_–HC) and (c) hollow carbon hosting sulfur (S@HC). Reproduced by permission.^73^ Copyright 2018, Springer Nature. (d) Aberration-corrected HAADF STEM of Co_SA_–HC. (e) Co XANES spectra of Co_SA_–HC, Co foil and Co phthalocyanine (CoPc). (f) Energy profiles for the reduction of lithium polyselenides on NC and Co–NC supports (insets: the optimized adsorption conformations of the intermediate species on the NC and Co–NC substrate). Energy profiles of the transformation of Li_2_Se clusters on NC (g) and Co–NC (h). (The insets are: the initial, transition, and final structures, respectively.) The brown, pink, green, blue, and cyan balls represent C, Se, Li, N and Co atoms, respectively. Reproduced by permission.^74^ Copyright 2020, Springer Nature.

Na–S batteries have competitive merit over Li–S batteries, *e.g.*, having specific energy density (760 W h kg^−1^ compared with 500–600 W h kg^−1^ for Li–S batteries), the larger natural abundance of sodium (more than 400 times the availability of lithium), and its relatively low cost.^75^ High-temperature Na–S batteries have been used for stationary energy storage for decades.^76^ However, the extra cost and high risks at high temperatures have hindered the further development of high-temperature Na–S batteries.^77^ Therefore, it is a promising approach to develop room-temperature sodium–sulfur (RT-Na–S) batteries. Compared with Li–S batteries, the problems (such as the shuttling effect, the volume change of sulfur, poor sulfur conductivity, and the formation of insoluble sulfides) have so-far resulted in inferior electrochemical performances for the RT-Na–S batteries. To overcome these challenges, the utilization of SACs has become an alternative method to restrain the shuttling effect and activate the sulfur activity. For example, sulfur cathode materials containing atomic cobalt-decorated hollow carbon nanospheres (S@Co_*n*_–HC) have been designed and prepared *via* calcination of hollow carbon spheres with CoCl_2_ precursors with a subsequent sulfur encapsulation step to prepare electrode materials for RT-Na–S batteries.^73^ DFT calculations (Fig. 8a) indicated that atomic cobalt could electro-catalyze the decomposition of Na_2_S_4_ by enhancing the binding energies of sodium polysulfides on single cobalt carbon supports compared with the pure carbon support. The resulting S@Co_*n*_–HC cathode materials have achieved excellent reversible capacity of 508 mA h g^−1^ at 100 mA g^−1^ after 600 cycles, which is much higher than that for S@HC (271 mA h g^−1^). This excellent electrochemical behaviour of S@Co_*n*_–HC is attributed to the atomic Co further enhancing the conductivity, electrocatalysing the sulfur conversion, and alleviating the dissolution of sodium polysulfides (Fig. 8b and c).

Due to the similarity between sulfur and selenium in the same group of the periodic table, selenium has been treated as a possible substitute cathode material for lithium–selenium (Li–Se) batteries due to its high theoretical volumetric capacity (3253 mA h cm^−3^) and high conductivity (1 × 10^−3^ S m^−1^).^78^ However, the dissolution issue of high-order lithium selenides (Li_2_Se_*x*_, *x* > 4) and large volume expansion during the charge/discharge processes in the Li_2_Se_*x*_ cathode materials lead to poor electrochemical performance and a low selenium utilization.^79^ Solutions to these problems are being demonstrated. Co single atoms have been applied to activate the selenium reactivity and immobilize selenium and polyselenides, achieving high rate capability and outstanding long-term cycling performance for Li–Se batteries.^74^ Co single atom/N-doped hollow porous carbon (Co_SA_–HC) was obtained from the heat treatment of MOFs. The aberration-corrected HAADF-STEM image reproduced in Fig. 8d provides strong evidence that single Co atoms have been homogeneously dispersed within Co_SA_–HC particles. The XANES results in Fig. 8e further reveal that the single Co atoms are positively charged, and the EXAFS data shows that isolated single Co atoms are atomically anchored on the carbon frameworks through the formation of Co–N_3_ and Co–N_4_ coordination moieties within the Co_SA_–HC particles. To further understand the enhancement of the reaction kinetics of the charge/discharge of these Se@Co_SA_–HC cathodes, first-principles calculations were conducted with results as depicted in Fig. 8f. The results indicate that the reaction rate for the reduction of Li_2_Se_2_ into Li_2_Se is noticeably increased during the discharge process by using single Co atom catalysts. The charging process data suggests that a smaller value than the calculated energy barriers for Li_2_Se decomposition (as shown in Fig. 8g and h) can be obtained through the provision of single Co atom catalysts.

### 3.3. Lithium–oxygen batteries

Lithium–oxygen (Li–O_2_) batteries have been treated as one of the most significant energy storage systems because of the theoretically high gravimetric energy density (∼3505 W h kg^−1^, based on O_2_ + 2Li^+^ + 2e^−^ ↔ Li_2_O_2_), stable discharge voltage, and long storage life.^80^ Regarding the discharge process in lithium–oxygen batteries, lithium ions can react with oxygen to form Li_2_O or Li_2_O_2_ inside the porous cathode materials through ORR. During the change process in lithium–oxygen batteries, decomposition of Li_2_O or Li_2_O_2_ and oxygen release can occur through OER.^81^ The issues of low coulombic efficiency, poor rate capability, and inferior cycling life have restricted the advancement of lithium–oxygen batteries because of the insoluble and insulating Li_2_O_2_.^82^ To address these problems, several electrocatalytic materials, such as noble metals, metal oxides, and sulfides, have been used to promote the electrochemical reactions during discharging and charging, leading to an overpotential decrease between ORR and OER.^83^

**Fig. 9:**
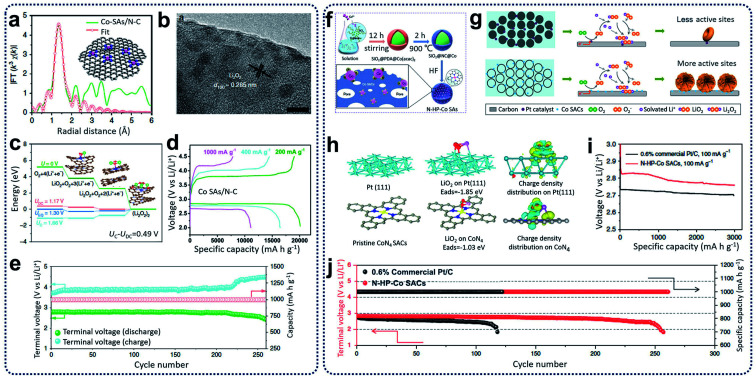
(a) EXAFS fitting curves in *R* space, inset showing the schematic model (the pink, blue, and gray balls stand for Co, N, and C, respectively). (b) *Ex situ* HRTEM image of fully discharged Co–SAs/N–C. (c) Calculated free energy diagrams for the discharge–charge reactions on the active surface of Co–SAs/N–C. (d) The discharge–charge curves at different current densities. (e) Cycling stability and terminal discharge–charge voltages of the Co–SAs/N–C electrode at 400 mA g^−1^ with a limited capacity of 1000 mA h g^−1^. Reproduced by permission.^84^ Copyright 2020, Springer Nature. (f) The synthesis procedures for N–HP–Co SACs. (g) Discharge mechanism of 0.6% commercial Pt/C and N–HP Co SACs in Li–O_2_ batteries. (h) Pristine and top views of the optimized structures with the corresponding binding energy of LiO_2_ on Pt (111) and CoN_4_ and the corresponding charge density distribution. Color code: platinum (green), carbon (grey), CoN_4_ (blue), and LiO_2_ (red). (i) First discharge curves of 0.6% commercial Pt/C and N–HP–Co SACs at a current density of 100 mA g^−1^ with a limiting specific capacity of 3000 mA h g^−1^. (j) Voltage *versus* cycle number on the discharge terminal of the Li–O_2_ cells with commercial Pt/C and N–HP–Co SACs. Reproduced by permission.^85^ Copyright 2020, Springer Nature.

Recently, SACs have been reported as active catalysts for Li–O_2_ batteries to yield a low charge overpotential, high reversible capacity, and good cycling stability.^84,85^ Yin and co-workers reported the synthesis of N-doped carbon nanosheet-supported isolated Co SACs (Co–SAs/N–C) through the pyrolysis of Zn-hexamine precipitates for Li–O_2_ batteries.^84^ It was found that single Co atoms with CoN_4_ configurations (Fig. 9a) in Co–SAs/N–C catalysts can improve the absorption ability for LiO_2_, leading to the homogeneous distribution of Li_2_O_2_ with particles size of 2–3 nm during ORR (Fig. 9b). Compared with the Co–SAs/N–C catalysts, large Li_2_O_2_ aggregates were generated in the control samples, which included Co nanoparticles trapped in N-rich carbon (Co–NPs/N–C) and N-doped carbon nanosheets (N–C). Through computational calculations, the Co–N_4_ moieties were shown to play an important role in the reduction of Li_2_O_2_ generation and oxidization overpotentials (see Fig. 9c). When tested as Li–O_2_ batteries, the Co–SAs/N–C electrodes achieved a high-rate discharge capacity (11 098 mA h g^−1^ at 1 A g^−1^) and an excellent cycling ability (260 cycles at 400 mA g^−1^) with an ultra-low charge/discharge polarization (0.40 V) (Fig. 9d and e). Additionally, Xu *et al.* prepared hollow N-doped porous carbon sphere-structured Co SACs (N–HP–Co) through hard-template methods (Fig. 9f).^85^ It was reported that during the discharge process in Li–O_2_ batteries, atomic Co sites in the N–HP–Co catalysts served as nucleation sites for the growth of uniform nanosheets of Li_2_O_2_ compared to commercial Pt/C, which adopted a toroidal morphology on the carbon surfaces (Fig. 9g). In the charging process, Li_2_O_2_ decomposition is kinetically more favourable and reversible for N–HP–Co catalysts through a one-electron pathway compared to commercial Pt/C catalysts with two-electron pathways (Fig. 9h). As a result, the N–HP–Co electrodes exhibited excellent cycling ability (261 cycles at a current density of 100 mA g^−1^ with a cut-off capacity of 1000 mA h g^−1^) and a high discharge capacity (∼14 777 mA h g^−1^ at a current density of 100 mA g^−1^) for the Li–O_2_ batteries (Fig. 9i and j).

### 3.4. Zinc–air batteries

Compared with the aforementioned lithium/sodium metal batteries and lithium/sodium–sulfur batteries, rechargeable Zn–air batteries with high theoretical specific energy density (1350 W h kg^−1^, excluding oxygen), inherent safety, environmental friendliness, and economic viability have broad application prospects for the consumer electronics market and portable devices. Differing from the lithium–oxygen batteries, Zn–air batteries have reliable stability to protect Zn anodes from water.^86^ However, the development of Zn–air batteries is still facing critical issues like lower energy efficiency (<65%) brought by the sluggish kinetics of ORR and OER at air cathodes and zinc dendrite generation on Zn anodes.^87^ Catalysts including precious metals, spinel oxides, carbon-based non-precious metal have all been tested in attempts to obtain: (1) excellent reaction kinetics at air electrodes with fast direct 4e^−^ pathways for the ORR and OER processes, as well as (2) excellent reaction stability. Even the most outstanding Pt-, Ir- or Ru-based catalysts still encounter obstacles, such as high cost and poor durability, for achieving and maintaining high-performance in Zn–air batteries. Recently, the introduction of SACs as bifunctional catalysts for the air electrodes of Zn–air batteries has shed special light on the research in this direction.^88,89^ SACs, benefitting from their homogeneous active sites, unique electronic structures, and high catalytic activity, display promising charging and discharging parameters when assembled as a cathode in rechargeable and solid-state Zn–air batteries. Enormous efforts have been devoted to boosting the catalytic kinetics of the catalysts by changing the absorption/desorption energies of the intermediates of the reaction steps, as well as promoting the transport of the intermediate species, aimed at further improving the performance of the Zn–air batteries assembled with these catalysts. Table 1 summarizes the performance reported in recent works on the primary, rechargeable, and solid-state Zn–air batteries using SACs, including parameters, such as open-circuit voltage (OCV), peak power density, specific capacity, and cycling stability.

**Table 1:** Performance of recently reported SACs in Zn–air batteries

Catalysts	Application	Open circuit voltage (OCV, V)	Peak power density (mW cm^−2^)	Specific capacity (mAh g_Zn_^−1^)	Cycling current density (mA cm^−2^)	Cycling stability	Ref.
Fe–N_*x*_–C	Rechargeable Zn–air battery	1.51	96.4	641	5	300 h	90
	Flexible all-solid-state Zn–air battery	1.49	—	—	1	120 h	
FeN_*x*_-PNC	Rechargeable Zn–air battery	1.55	278	—	10	55 h	92
Fe-NSDC	Rechargeable Zn–air battery	1.53	225.1	740	4	400 cycles	91
Fe-OES	Rechargeable Zn–ir battery	∼1.5	186.8	807	5	400 cycles	93
Fe-NCC	Primary Zn–air battery	1.52	—	739	—	—	94
Fe SAs/N–C	Primary Zn–air battery	—	225	636	—	—	95
	Rechargeable Zn–air battery	—	—	—	10	260 h	
FeN_4_ SAs/NPC	Rechargeable Zn–air battery	—	232	—	2	108 cycles	96
Fe/N–G-SAC	Rechargeable Zn–air battery	—	120	—	10	240 cycles	97
Fe–N–C-700	All-solid-state flexible Zn–air battery	1.42	70	663	1	—	98
Co–N_*x*_/C NRA	Rechargeable Zn–air battery	1.42	193.2	275	50	80 h	99
CoN_4_/NG	Rechargeable Zn–air battery	1.51	—	730	10	100 h	88
	All-solid-state Zn–air battery	—	28	—	1	6 h	
Co–Co_3_O_4_@NAC	Rechargeable Zn–air battery	1.449	164	721	10	35 h	100
					5	35 h	
Co–N, B-CSs	Rechargeable Zn–air battery	1.43	100.4	—	5	14 h	101
	All-solid-state Zn–air battery	1.345	—	—	2	∼22 h	
NC–Co SA	Rechargeable Zn–air battery	—	—	—	10	570 cycles	102
	Flexible solid-state Zn–air battery	1.411	20.9		10	∼2500 min	
SCoNC	Rechargeable Zn–air battery	1.49	194	690	5	60 cycles	103
Ni, N co-doped np-graphene	All-solid-state Zn–air battery	1.35	83.8	—	2	258 cycles	104
CoNi–SAs/NC	Rechargeable Zn–air battery	1.45	101.4	750	5	95 cycles	105
Zn–N–C	Rechargeable Zn–O_2_ battery	—	179	683	—	—	106
Zn/CoN–C	Rechargeable Zn–air battery	—	230	—	5	100 000 s	107
(Zn,Co)/NSC	Primary Zn–air battery	1.5	150	—	—	—	108
	Solid-state Zn–air battery	1.56	15	—	—	—	
Ag–MnO_2_	Rechargeable Zn–air battery	—	273.2	—	10	3200 cycles	109
Mn/C–NO	Rechargeable Zn–air battery	—	120	—	20	20 000 s	110
CuSA@HNCN_*x*_	Rechargeable Zn–air battery	1.51	212	806	10	300 h	111
	Solid-state flexible Zn–air battery	1.51	202	793	25	250 h	
Cu ISAS/NC	Primary Zn–air battery	—	280	∼736	—	—	112

**Fig. 10:**
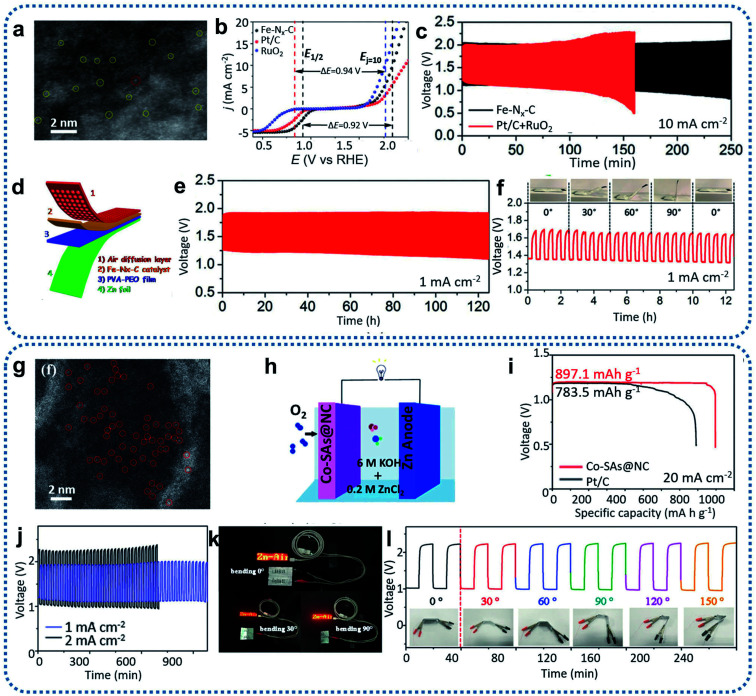
(a) Aberration-corrected HAADF-STEM image of Fe–N_*x*_–C. (b) Overall polarization curves of Fe–N_*x*_–C, commercial Pt/C, and commercial RuO_2_ in 0.1 M KOH. (c) Cycling test at a current density of 10 mA cm^−2^ with the Fe–N_*x*_–C and Pt/C + RuO_2_ mixture catalysts, respectively. (d) Schematic illustration of the all-solid-state Zn–air battery. (e) Cycling performance of the all-solid-state Zn–air battery tested at a current density of 1 mA cm^−2^. (f) Foldability test of an all-solid-state Zn–air battery bent to different angles from 0° to 90° at a current density of 1 mA cm^−2^. Reproduced by permission.^90^ Copyright 2019, Wiley-VCH. (g) HAADF-STEM images of Co–SAs@NC. (h) Schematic illustration of an aqueous Zn–air battery. (i) Discharge curves of the primary Zn–air battery catalyzed by Co–SAs@NC and Pt/C at 20 mA cm^−2^. (j) Discharge–charge performance of the flexible Zn–air battery. (k) Photographs of an LED screen light by two batteries connected in series based on the Co–SAs@NC catalyst. (l) Cycling performance of the battery with a Co–SAs@NC cathode at different bending angles and 2 mA cm^−2^ current density. Reproduced by permission.^113^ Copyright 2019, Wiley-VCH.

Typically, single-atom Fe-based catalysts were successfully prepared based on MOF coating and high-temperature thermalization technology (Fig. 10a).^90^ After conducting electrochemical tests on a basic three-electrode system, the prepared Fe–N_*x*_–C catalyst exhibited excellent ORR and OER catalytic performance and long-term stability (Fig. 10b). Therefore, a Fe–N_*x*_–C-based primary liquid Zn–air battery was first assembled to examine the discharging performance. The resulting Fe–N_*x*_–C-based Zn–air batteries delivered superior performance with a high OCV of 1.51 V, a peak power density of 96.4 mW cm^−2^, and a discharge specific capacity as high as 641 mA h g^−1^. All these battery parameters are better than those of Zn–air batteries assembled with the benchmark Pt/C (1.45 V, 82 mW cm^−2^, 545 mA h g^−1^). Subsequently, long-term galvanostatic cycling tests were conducted by Fe–N_*x*_–C-assembled rechargeable Zn–air batteries, which achieved more than 250 h of stable operation at a cycling current density of 10 mA cm^−2^ (Fig. 10c). The round-trip efficiency decayed from 56.2% to 39.9%, but this is still 14.1% higher than Zn–air batteries assembled using commercial Pt/C and RuO_2_ (57.3%, 25.8%, respectively). Furthermore, the Fe–N_*x*_–C-based flexible all-solid-state Zn–air battery (Fig. 10d) displayed a high OCV of 1.49 V and ran stable discharge–charge cycling for more than 120 h (Fig. 10e). No significant changes were observed in the discharging voltage, charging voltage, or overpotential value of the flexible Zn–air batteries after folding to different bending angles from 0° to 90° (Fig. 10f). This indicated the materials' development potential for flexible portable devices. In the following work, to enhance the performance of traditional Fe–N–C catalysts, sulfur was doped through a one-step pyrolysis method.^93^ S, as a charge-forming dopant, could regulate the electronic structure of the Fe–N_*x*_ sites to optimize the intermediate OH^−^ adsorption and O_2_ desorption. Sulfur doping of Fe–NSDC improved the intrinsic activity of the prepared Fe-NSDC catalysts for ORR and OER. Rechargeable Zn–air batteries assembled with N, S-codoped Fe-NSDC exhibited an excellent OCV of 1.53 V, a power density of 225 mW cm^−2^, and a specific capacity as high as 740 mA h g^−1^.

Apart from Fe-based SACs, Co-based SACs with high catalytic activity are also being widely investigated as bifunctional catalysts to be utilized in Zn–air batteries. For example, Co-based SACs derived from ZIFs were successfully synthesized through precisely controlling the molar ratio of Co : Zn (Fig. 10g).^113^ The introduction of Zn^2+^ into the precursor effectively led to an atomic dispersion of the cobalt species. The prepared Co–SAs@NC catalyst with isolated Co sites, excellent conductivity, rich pore structure, and large specific surface area displayed remarkable ORR and OER performance. For practical testing, it was assembled in a prototype Zn–air battery to determine the recharging ability. The assembly diagram of this Zn–air battery is shown in Fig. 10h. The final Zn–air battery consisted of a zinc anode with a Co–SAs@NC catalyst cathode, and concentrated alkaline electrolyte containing 6.0 M KOH and 0.2 M ZnCl_2_. Such Co–SAs@NC-based primary Zn–air batteries delivered an OCV of 1.46 V and a maximum power density of 105 mW cm^−2^, approximating the parameters of commercial Pt/C (1.41 V, 110 mW cm^−2^). Furthermore, it achieved a much higher discharge specific capacity of up to 897 mA h g^−1^ at 20 mA cm^−2^ compared to 783 mA h g^−1^ for Pt/C (Fig. 10i). Co–SAs@NC-based rechargeable Zn–air batteries have delivered more than 250 h of stable performance with a charge/discharge overpotential of 0.85 V after cycling at a current density of 10 mA cm^−2^ (Fig. 10j). Moreover, the Co–SAs@NC catalyst has been used to assemble a flexible solid-state Zn–air battery. The flexible Zn–air battery displayed an OCV of 1.40 V, and was able to function well for 1000 min at 1 mA cm^−2^, 700 min at 2 mA cm^−2^. Two flexible solid-state Zn–air batteries based on the Co–SAs@NC connected in series easily powered a red light-emitting diode (LED) mini-display (Fig. 10k), and the operation remained stable at different bending angles from 0° to 150° (Fig. 10l), indicating the capability for portable electronic devices. Moreover, outstanding charge–discharge capacity and power density have been demonstrated in CoN_4_/NG- and Co–Co_3_O_4_@NAC-assembled Zn–air batteries, respectively.^88,100^

**Fig. 11:**
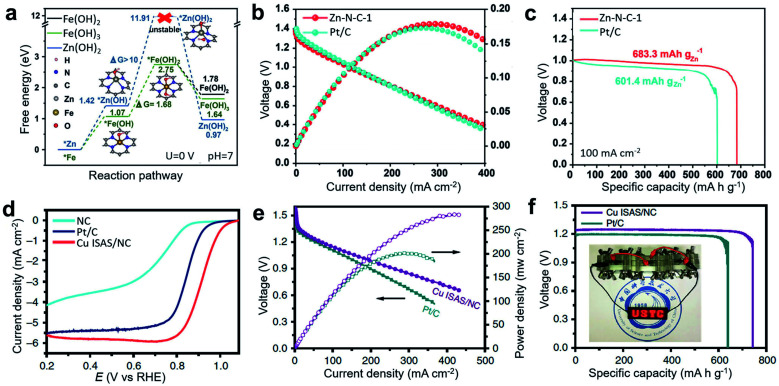
(a) Free-energy diagrams for Zn(OH)_2_, Fe(OH)_2_, and Fe(OH)_3_ during the metal corrosion process, as based on the M–N_4_ (M = Zn/Fe) structure. (b) Discharging polarization and the corresponding power density curves of a primary Zn–air battery at a scan rate of 5 mV s^−1^. (c) Discharge curves of the Zn–air battery at 100 mA cm^−2^ discharge rate. Reproduced by permission.^106^ Copyright 2019, Wiley-VCH. (d) Linear sweep voltammetry (LSV) curves of NC, Cu ISAS/NC and Pt/C catalysts. (e) Polarization and corresponding power density plots. (f) Specific capacity at 50 mA cm^−2^. (Inset: a photograph showing light-emitting diode panel powered by three Cu ISAS/NC-based Zn–air batteries). Reproduced by permission.^112^ Copyright 2019, Springer Nature.

In addition to the commonly studied Fe-based and Co-based SACs, many other metals, such as Zn, Ni, Mn, and Cu have been developed into bifunctional catalysts for Zn–air batteries. Among them, Zn is very promising because metallic Zn has a filled d orbital (3d_10_4s_2_), so zinc ions are hard to oxidize into a higher valence state, providing scope for protecting electrodes and separator membranes. Therefore, Zn-based catalysts have considerable advantages for maintaining the stable operation of Zn–air batteries. To effectively improve the catalytic performance of ORR and OER, precursors containing ZnCl_2_ and *o*-phenylenediamine (*o*PD) have been converted into stable Zn–N_*x*_ sites to synthesize Zn SACs (Zn–N–C-1) with a high Zn loading of 2.06 at% by controlling the heating rate at 1° min^−1^ at a high pyrolysis temperature.^106^ Compared with Zn–N–C catalysts with lower loading, Zn–N–C-1 exhibited obvious ORR advantages in both acidic and basic systems. In addition, Zn–N–C-1 catalysts had comparable catalytic performance and better stability to Fe–N–C-1 prepared under the same conditions. According to the DFT calculation results, the transformation of a *Zn metal site to *Zn(OH) and further to *Zn(OH)_2_ at a Zn–N_4_ site was more difficult than the corresponding activation of Fe at a Fe–N_4_ site, confirming the better stability of the Zn-based catalyst during battery operation (Fig. 11a). The prepared Zn–N–C catalyst achieved high activity and long-term stability under basic conditions for ORR electrochemical tests. When assembled in a primary Zn–O_2_ battery, Zn–N–C-1 catalysts as an air electrode delivered a high peak power density of 179 mW cm^−2^ and a specific capacity of 683 mA h g_Zn_^−1^ (Fig. 11b and c), which is higher than the performance of commercial Pt/C-assembled Zn–O_2_ battery (173 mW cm^−2^, 601 mA h g_Zn_^−1^). Cu-based SACs have also been utilized in Zn–air batteries. For example, the commercially available Cu_2_O was used as a direct source of single Cu atoms on an N-doped carbon (NC) substrate through a simple gas-transport strategy.^112^ This method is simple and feasible to apply in mass production. The synthesized catalyst has excellent ORR performance with a half-wave potential as high as 0.92 V (*vs.* RHE) (Fig. 11d). For practical application, primary Zn–air batteries assembled with the prepared Cu ISAS/NC catalyst showed a peak power density as high as 280 mW cm^−2^ and a specific capacity of ∼736 mA h g^−1^, superior to that of the commercial Pt/C-based catalyst (200 mW cm^−2^, ∼640 mA h g^−1^) and other reported catalyst-based primary Zn–air batteries (Fig. 11e and f).

To sum up, SACs with excellent ORR (or bi-functional ORR and OER) performance can be utilized for rechargeable Zn–air batteries and flexible solid-state Zn–air batteries. To better understand the electrochemical behaviour of Zn–air batteries, the catalytic mechanism bifunctionalities of SACs should be further studied by theoretical and experimental investigations. Moreover, the oxidation and corrosion resistance of the SACs (especially carbon-based SACs) faced when assembling them into Zn–air batteries need to be further enhanced.

## 4. Conclusions and outlook

Great innovations have been made in the advancement of SACs with controllable functionalities, well-designed morphologies, and adjustable porosities. This review summarizes the recent progress on SACs from their synthetic and modification strategies, especially for rechargeable batteries. Various SACs with different active single atoms and matrices have been designed and investigated. The unsaturated and low-coordinated active sites with high surface energy within SACs can promote electrochemical reactions and achieve superior battery performances with high energy density, high current rates, and long cycling life. Many challenges are needed to be addressed, including the well-controlled and large-scale synthesis of SACs, SACs with high single atom loading, SACs with dual or multi-single atoms, and the in-depth understanding of electrochemical mechanisms of SACs. The specific challenges and efforts are presented below.

SACs with well-defined structures and unsaturated coordination can be achieved through careful synthetic strategies, including the spatial confinement method, defect design method, impregnation, and coprecipitation method, coordination engineering method, and other ingenious methods. However, SACs usually present a range of different catalytic activities because of the complicated coordination environments and electronic structure of their active components. Therefore, it is a great challenge to precisely and controllably synthesize SACs with regulated coordination number and atomic distributions on (or throughout) substrates. In addition, it is important to produce SACs by synthesis methods that are suitable for large-scale production. Most significantly, the synthesis of SACs usually involves preparing precursors, such as MOFs, molecular sieves, organics, or polymers, with subsequent calcination process, which may entail high production costs, harsh reaction conditions, and generation of hazardous byproducts (*e.g.*, vapours when transforming precursors into final substrates). It is highly desired to achieve SACs *via* “green” technology for large-scale manufacturing.

Additionally, it is well-recognized that exploring SACs with high mass loading and uniform dispersion to enhance the electrochemical performances of batteries is a vital challenge to be solved. The concentration of single atoms within SACs has to be delicately controlled because single atoms easily aggregate under high mass loading. Such aggregation within catalysts can reduce the catalytic activities. In particular, high levels of aggregation can induce nonuniform active metal nucleation and prompt phase separation between metal aggregates and the supporting substrate, leading to low coulombic efficiency, low rate capability, and short cycling life. Through carefully choosing metal precursors and matrices such as two-dimensional materials (graphitic carbon nitride and graphene) with engineered defects by enhancing the coordination ability between metal atoms and matices, SACs with high mass loading can be achieved. However, it is still hard to predict the compatibility between the metal species and matrix materials to achieve SACs with high mass loading. With the development of theoretical calculation packages, first principles calculations can provide an effective approach to understand and forecast SACs properties with high mass loading and well-defined structures for various rechargeable batteries.

To further enhance electrocatalytic processes, including the adsorption/desorption and activation behaviours of reactants and products in battery systems, dual versions of single-atom catalysts within SACs should also be considered for their possible synergistic effects. Dual atoms can potentially further enhance atom utilization, reduce energy barriers and improve catalytic efficiency. Because of the high surface energy, single atoms within SACs can aggregate into clusters or even nanoparticles during the electrochemical reactions, leading to the irreversible decrease of the catalytic activity. Single atoms can also be leached out from SACs with the loss of active components, resulting in the poor efficiency of electrochemical reactions. Therefore, it is required to enhance the interaction between the single atoms and their support substrates to attain high chemical and structural stability through physical confinement and chemical binding.

It is crucial to comprehensively understand the co-relationships between the chemical/physical properties of the SACs and their catalytic performances. Therefore, it is desirable to use advanced techniques such as aberration-corrected HAADF-STEM, XANES, and EXAFS for accurate characterization of SACs. An in-depth understanding of single-atom catalysts in terms of their local electronic structure, their coordination environment, and bonding distance can determine and elaborate their catalytic activities in rechargeable batteries.

To gain further understanding of electrochemical mechanisms of SACs in rechargeable batteries, *in situ* and *ex situ* characterizations should be conducted together with the assistance of theoretical calculations. For example, *in situ* TEM technologies can be applied to investigate the detailed morphological evolution (nucleation and growth) of polysulfides when SACs are used during electrochemical reactions in the proposed Li/Na sulfur batteries. In addition, *in situ* XRD can be used to determine the phase structure during the discharging and charging processes. DFT calculations provide detailed insight into the adsorption/desorption behavior of reaction intermediates such as polysulfides on the interfaces of SACs within lithium/sodium–sulfur batteries. Thus, the *in situ* characterizations and DFT calculations can elucidate the mechanisms of reactions fostered by SACs.

In conclusion, precisely controllable synthesis is critical to achieve high-performance single-atom catalysts. The application of SACs could boost the development of new generation rechargeable batteries such as lithium/sodium metal batteries, lithium/sodium–sulfur batteries, lithium–oxygen batteries, and zinc–air batteries.

## Author contributions

H. Tian and A. L. Song contributed equally to this work. H. Tian, A. L. Song, H. Liu and G. X. Wang conceived and designed the structure of the review. H. Tian and A. L. Song collected the papers related to the topic of the review. H. Tian and A. L. Song co-wrote the paper with input from H. J. Tian, J. Liu, G. J. Shao, H. Liu and G. X. Wang. The paper was revised by all authors.

## Conflicts of interest

The authors declare no conflict of interest.
